# Self- and proxy-reported impaired social interaction in young adults with simple congenital heart defects

**DOI:** 10.3389/fped.2023.1165820

**Published:** 2023-09-06

**Authors:** Sara Hirani Lau-Jensen, Benjamin Asschenfeldt, Lars Evald, Vibeke E. Hjortdal

**Affiliations:** ^1^ Department of Clinical Medicine, Faculty of Health and Medical Science, University of Copenhagen, Copenhagen, Denmark; ^2^ Department of Cardiothoracic Surgery, Copenhagen University Hospital, Copenhagen, Denmark; ^3^ Department of Clinical Medicine, Faculty of Health, Aarhus University, Aarhus, Denmark; ^4^ Hammel Neurorehabilitation and Research Centre, Hammel, Denmark

**Keywords:** social interaction, simple congenital heart defect, atrial septal defect, ventricular septal defect, young adult

## Abstract

**Background:**

Simple Congenital Heart Defects such as septal defects constitute a large proportion of Congenital Heart Defects. New research has demonstrated more co-morbidities than previously thought. In particular, co-morbidities involving neurocognitive, psychiatric, and social difficulties have been described. Neurocognitive and psychiatric morbidities affect social interaction. Social interaction is important in everyday social life (education, work life, family life). In this study, we investigated social interaction through self- and proxy-answered Social Responsiveness Scale 2 (SRS-2) in young adults with simple Congenital Heart Defects and compared their social interaction profile to healthy matched controls.

**Methods:**

We included a total of 80 patients with either atrial or ventricular septal defect (age 26.6 years) and 38 heart-healthy, age, sex, and ISCED educational matched controls (age: 25.3 years). A close relative proxy from each participant took part in the study as well. All participants answered the Social Responsiveness Scale 2 (SRS-2) (*n* = 225). Our primary and secondary outcomes were the SRS-2 Total score and the SRS-2 sub-scores.

**Results:**

In the Congenital Heart Defects group, 31.3% had a Total score above 60 compared to 7.9% in the control group (*p* = 0.005, RR = 3.96). The participants with a septal defect had a higher Total score (52.5 vs. 45.5, *p* = 0.004), a higher Social Cognition sub-score (55.0 vs. 47.0, *p* = 0.0004), and a higher Social Motivation sub-score (50.0 vs. 45.0, *p* = 0.003) than the heart-healthy participants. We found no difference between the two groups regarding the sub-scores of Social Awareness and Social Communication. A multiple linear regression model showed that the variable that explained most of the variation in Total Score was having a previously diagnosed psychiatric disorder.

**Conclusion:**

We found that young adults with atrial or ventricular septal defects have a fourfold increased risk of social interaction difficulties compared to heart-healthy peers. They have a social interaction profile, with difficulties in social cognition and social motivation, and preserved social awareness and social communication. Psychiatric morbidity explained most of the variation in social interaction problems. As social difficulties and psychiatric morbidities are intertwined, social interaction difficulties could be an indication of already underlying psychiatric morbidities or a risk factor for future psychiatric morbidity.

## Introduction

Congenital Heart Defect (CHD) is the most common birth defect (5.5–8/1,000 all births) (1). Advancements in treatment and improved survival rates have shifted attention toward understanding the long-term consequences of CHD (2–4). As CHD is a heterogeneous group of birth defects it can be grouped into simple [involving a single defect or a combination of minor defects with minimal impact on overall heart function (if treated), e.g., atrial septal defects or ventricular septal defects], moderate (more complex abnormalities that affect the heart function to a greater extent e.g., tetralogy of Fallot), and complex CHD (very complex abnormalities that affect heart function and requires extensive medical management and lifelong follow-up e.g., hypoplastic left heart syndrome) based on the European Society of Cardiology Guidelines (5). Long-term neurocognitive and psychiatric morbidities are not only found in patients with complex CHDs (6–9) but also in patients with simple CHDs such as atrial and ventricular septal defects (6–8). Patients with simple CHDs further have a lower educational level and an increased risk of unemployment (9, 10).

Social function is the skill to understand social situations and to use and interpret social signals correctly (11). These abilities include sensory perception of social cues, interpreting these cues, and effectively communicating through socially expressive language and body language (12).

Difficulties in social function, including theory of mind difficulties, are found in children and adolescents born with different types of more complex CHD (11, 13, 14).

Social interaction is the most visually prominent aspect of social function. When an individual experiences challenges in social interaction, it is noticeable not only to the individual themselves but also to those surrounding them. Werninger et al. investigated a group of children who had undergone open heart surgery for a variety of CHD with the majority being of moderate to severe complexity. They found that the areas of social interaction affected were “social cognition”, “social motivation”, “social communication” and “repetitive behavior”, but not “social awareness” (14). Less is known about social interaction in patients with a simple CHD. Impaired social interaction has been described in a group of young adults with simple CHD (ASD and VSD) (7), but to our knowledge the profile of these social impairments is unknown.

We wanted to clarify the potential social interaction difficulties experienced by these patients. We investigated social interaction [though self and proxy answered Social Responsiveness Scale 2 (SRS-2)] in young adults with simple CHD. We compared their social interaction profile to healthy matched controls. We hypothesized that these young adults with simple CHD have more social interaction difficulties than heart-healthy peers. We also hypothesized that they would have a social interaction profile aligned with the one described by Werninger et al. in a mixed group of CHD patients involving “social cognition”, “social motivation” and “repetitive behavior”.

## Methods

The study is approved by the Committee on Biomedical Research Ethics from the Danish Central Regional (chart: 1-1072-233-17) and the Danish Data Protection Agency (chart: 2012-58-006). The study is registered on clinicaltrials.gov (identifier: NCT03871881) and it complies with the Declaration of Helsinki (the World Medical Association, 2013). Written informed consent prior to enrollment was obtained from all participants. The data supporting the findings of this study are available from the corresponding author upon reasonable request.

### Study population

We included patients older than 18 years of age with either an isolated atrial septal defect (ASD) or an isolated ventricular septal defect (VSD). Patients were recruited from a pool of patients diagnosed and treated at Aarhus University Hospital in the years between 1990 and 2000. Heart-healthy participants in the control group were recruited through flyers and announcements on the internet. Participants from both groups were excluded if they had either a known syndrome, e.g., Down syndrome or 22q11ds, recent head trauma, a previous stroke, ongoing pregnancy, or lack of sufficient Danish language skills. Participants in the control group were matched to both the ASD and the VSD group on age, sex, and educational level [International Standard Classification of Education ISCED (15)]. ISCED primary educational level corresponds to the completion of primary education (10–11 years), ISCED secondary educational level to the completion of secondary education (+2–3 years), and ISCED tertiary education refers to completed education beyond the secondary level.

A homogeneous group of doctors (anesthetists, cardiologists, and cardiac surgeons) from Aarhus University Hospital diagnosed and treated all patients in this study in the years between 1990 and 2000.

A close relative (proxy) from each participant participated in the study as well. The participants were informed that the proxy should be someone with good all-around knowledge about the participant, e.g., a parent, spouse, or sibling. The proxy was asked to answer the same questionnaires as the participants in the study.

All participants answered the Social Responsiveness Scale 2 (SRS-2) (total number of participants including patients, controls, and relatives = 225).

Participants from a previous study by Asschenfeldt et al. (7) constitute a subgroup of the patients in this study. The study by Asschenfeldt et al. did not include patients with percutaneously closed ASD or patients with still open ASD. For further detailed information on enrollment/recruitment procedures please refer to this study.

### Study set-up

Participants underwent a battery of neuropsychological tests by trained and experienced research assistants (supervised by LE). As part of this neuropsychiatric test participants underwent an IQ test [Wechsler Adult Intelligence Scale, 4th Edition (WAIS-IV)] (16) and the Read the Mind in the Eye test (RMET). Participants also answered self-reported questionnaires about social interaction [Social Responsiveness Scale 2 (SRS-2)]. Participants underwent a medical history interview also conducted by trained research assistants.

### SRS-2

The Social Responsiveness Scale 2 is a questionnaire designed to evaluate social interaction. It is composed of 65 questions and can differentiate social interaction into five sub-scores (Social Awareness, Social Cognition, Social Communication, Social Motivation, and Limited Interests and Repetitive Behavior). Results are reported as standardized *T*-scores (M = 50, SD = 10) based on normative data from the SRS-2 manual (12). A higher score indicates more difficulties.

The SRS-2 Total Score gives an overview of the degree of the overall social interaction difficulties.

The Social Awareness sub-score represents the ability to pick up social signals. The Social Cognition sub-score represents the ability to understand and correctly interpret these social signals once they are picked up. The Social Communication sub-score represents expressive social communication. The Social Motivation sub-score represents the degree of motivation the person has for engaging in social behavior (including social anxiety and empathy) (12). The Limited Interest and Repetitive Behavior sub-score represent challenges known to be a part of Autism Spectrum Disorder.

A Total Score above 60 (16th percentile) indicates a clinically significant lack of social abilities which can lead to difficulties in everyday social interactions. A Total Score above 60 is often related to social interaction difficulties seen in combination with psychiatric disorders other than Autism Spectrum Disorder. A Total Score above 85 is often related to social interaction difficulties seen in combination with Autism Spectrum Disorder (12).

The SRS-2 questionnaire consists of both a self-report questionnaire and a proxy-report questionnaire.

### Statistics

Our main outcome was a comparison of Total Scores above 60 and above 85 between the groups (control vs. CHD). This comparison was made with a chi-square test or a Fisher's Exact test if appropriate. Other outcomes included the between-group comparison (control vs. CHD) of sub-scores to evaluate the group profile of social interaction and the comparison between the self- and proxy-reports. A *post-hoc* analysis of both the Total Score and sub-scores comparing surgically closed ASD and percutaneously closed ASD was performed. Data is presented as medians with interquartile range or mean with SD, where appropriate. Group comparisons of non-parametric data were made with either Mann–Whitney *U*, Willcox Sign Rank test, or Median test. Parametric data was compared with either Welch's *T*-test or Student's *T*-test. A robust multiple regression model is made with Total Score as outcome and group, sex, previous psychiatric diagnosis, IQ, and Read the Mind in the Eye test (RMET) scores as variables. The multiple regression model was analyzed with each variable alone and all together in one block. Variables were chosen by prior knowledge about variables affecting social interaction skills (14, 17, 18). Previous psychiatric diagnosis, IQ, and RMET scores were chosen as psychological risk factors. CHD represented by group (control vs. CHD) and sex were chosen as medical risk factors.

*Post-hoc* analyses of the relationship between SRS-2 Total Score and the number of prior psychiatric diseases were made.

### Sample size justification

The sample size was calculated from the main outcome of the previously published study by Asschenfeldt et al. (7). A *post-hoc* calculation found that we would be able to detect a group difference in the SRS-2 Total Score of 3.66 with the sample size (*n* = 118) that we have, with a power of 80% and a significant level of 0.05. As the population norm given by the SRS-2 manual is 50 with an SD of 10, a smaller difference in the SRS-2 Total Score between the two groups may not be clinically relevant to detect.

## Results

We included a total of 80 patients with CHD (32 surgically closed VSDs, 34 surgically closed ASDs, 10 percutaneously closed ASDs, and 4 still open ASDs). A total of 38 heart-healthy, age, sex, and ISCED educational-matched participants were included as a control group.

The two groups of participants were comparable regarding sex, age, BMI, and educational level. More participants in the CHD group had a psychiatric diagnosis and three had a diagnosis of Autism Spectrum Disorder compared to none in the control group (Table 1).

**Table 1:** Demograhics.

Demographics	CHD (*N* = 80)	Control (*N* = 38)
Sex (*n*, %)		
Male	23 (28.8%)	13 (34.2%)
Female	57 (71.3%)	25 (65.8%)
Age		
Mean (SD)	26.6 (5.9)	25.3 (4.5)
BMI		
Mean (SD)	24.6 (4.9)	22.8 (3.2)
Education (*n*, %)		
ISCED^a^ primary education	3 (3.8%)	0 (0%)
ISCED^a^ secondary education	57 (71.3%)	25 (65.8%)
ISCED^a^ tertiary education	20 (25.0%)	13 (34.2%)
Any psychiatric diagnosis (*n*, %)		
Yes	34 (42.5%)	5 (13.2%)
Autism spectrum disorder (*n*, %)		
Yes	3 (3.8%)	0 (0%)
Informant relationship (*n*, %)		
CHD-informant (*n* = 74), information on all		
Control-informant (*n* = 33), information on all		
Parent	43 (53.8%)	11 (28.9%)
Spouse, partner	21 (26.3%)	10 (26.3)
Sibling	6 (7.5%)	8 (21.1%)
Friend	0 (0%)	2 (5.3%)
Unanswered	1 (1.3%)	0 (0%)
Duration of relation (years)		
Mean (SD)	18.9 (9.5)	16.9 (10.3)

^a^ International Standard Classification of Education ISCED (15).

### SRS-2 total score

**Figure 1:**
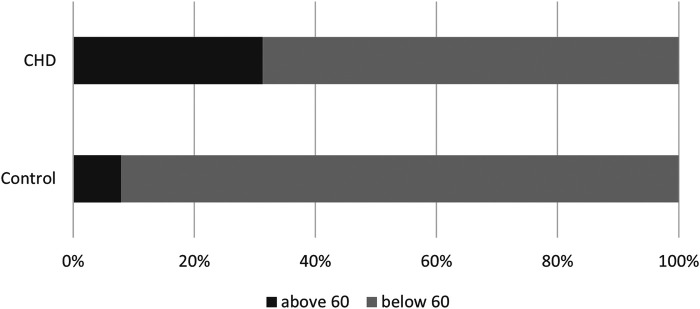
SRS-2 total score above 60 (self-reported).

In the CHD group, 31.3% had a Total Score above 60 compared to 7.9% of the participants in the control group (*p* = 0.005) (Figure 1). The participants in the CHD group had an increased/fourfold relative risk of having an elevated Total Score compared to the participants in the control group (RR = 3.96).

We found that 3.6% of participants from the CHD had a Total Score above 85 compared to 0% in the control group (*p* = 0.6).

The CHD group had a higher Total Score compared to the control group (*p* = 0.004) (Table 2).

**Table 2:** Self-reported SRS-2 scores.

	CHD (*N* = 80)	Control (*N* = 39)	CHD vs. control
Total *T*-score	52.5 (45.0–65.3)	45.5 (40.0–50.8)	0.004
Social awareness	49.0 (41.0–58.0)	45.0 (41.0–56.0)	0.1
Social cognition	55.0 (44.0–67.0)	47.0 (42.0–53.0)	0.004
Social communication	52.5 (44.0–63.0)	44.0 (39.0–51.5)	0.1
Social motivation	50.0 (45.0–60.5)	45.00 (40.0–50.0)	0.03
Limited interest and repetitive behavior	53.0 (46.0–63.0)	44.0 (40.0–51.0)	0.004

Benjamini-Hochberg correction, false discovery rate 0.05. Non-parametric median test.

### SRS-2 sub-scores

We performed an analysis of the different sub-scores to characterize the social interaction profile found in the CHD group compared to the control group.

Three out of five of the sub-scores (cognitive, motivation, and limited interest and repetitive behavior) were higher in the CHD group compared to the control group (Table 2). We found no significant difference between the two groups for the Social Awareness and for the Social Communication sub-score.

### Multiple regression

In a multiple linear regression model, we found that the variable that explained most of the variation in Total Score is having a previously diagnosed psychiatric disorder (Table 3).

**Table 3:** Robust linear regression.

Variables	Outcome					
	Total score	Total score	Total score	Total score	Total score	Total score
Group	−7.96* (1.37)					−3.30 (2.28)
Diagnosed psychiatric disorder		12.87 (2.15)*				10.27 (3.32)*
Sex			−1.77 (2.40)			−0.88 (2.12)
IQ				−0.22 (0.08)*		−0.09 (0.08)
RMET					−0.54 (0.33)	−0.24 (0.32)

Robust linear regression, Estimate (std. error).

Group ref = control, Diagnosed psychiatric disorder ref = Yes, Sex ref = Male.

* *P*-value <0.05.

**Figure 2:**
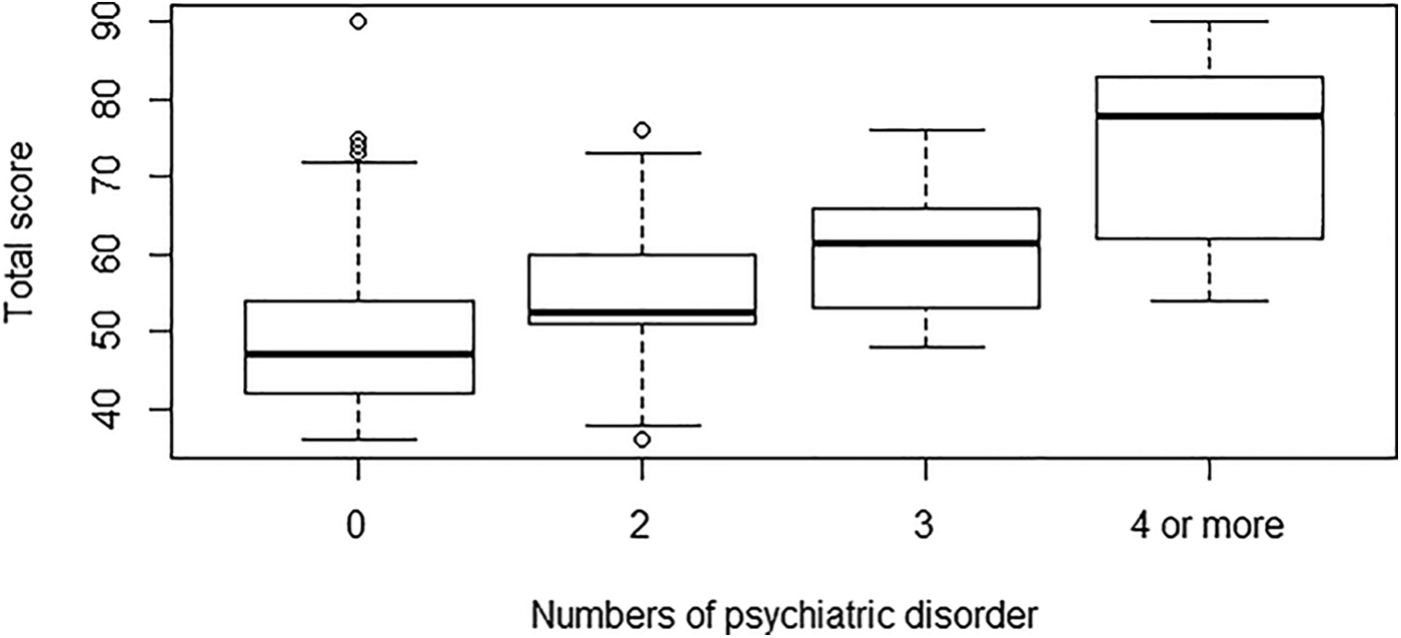
Boxplot of SRS-2 total score by numbers of diagnosed psychiatric disorders (all participants).

When we divided the participants by the number of psychiatric diagnoses they have received prior to the study (with the last category being “4 or more” due to a small number of participants with more diagnoses than 4), we found a trend towards increasing Total Score by increasing number of diagnosed psychiatric morbidities (Figure 2).

### Percutaneous procedure vs. surgical procedure

**Figure 3:**
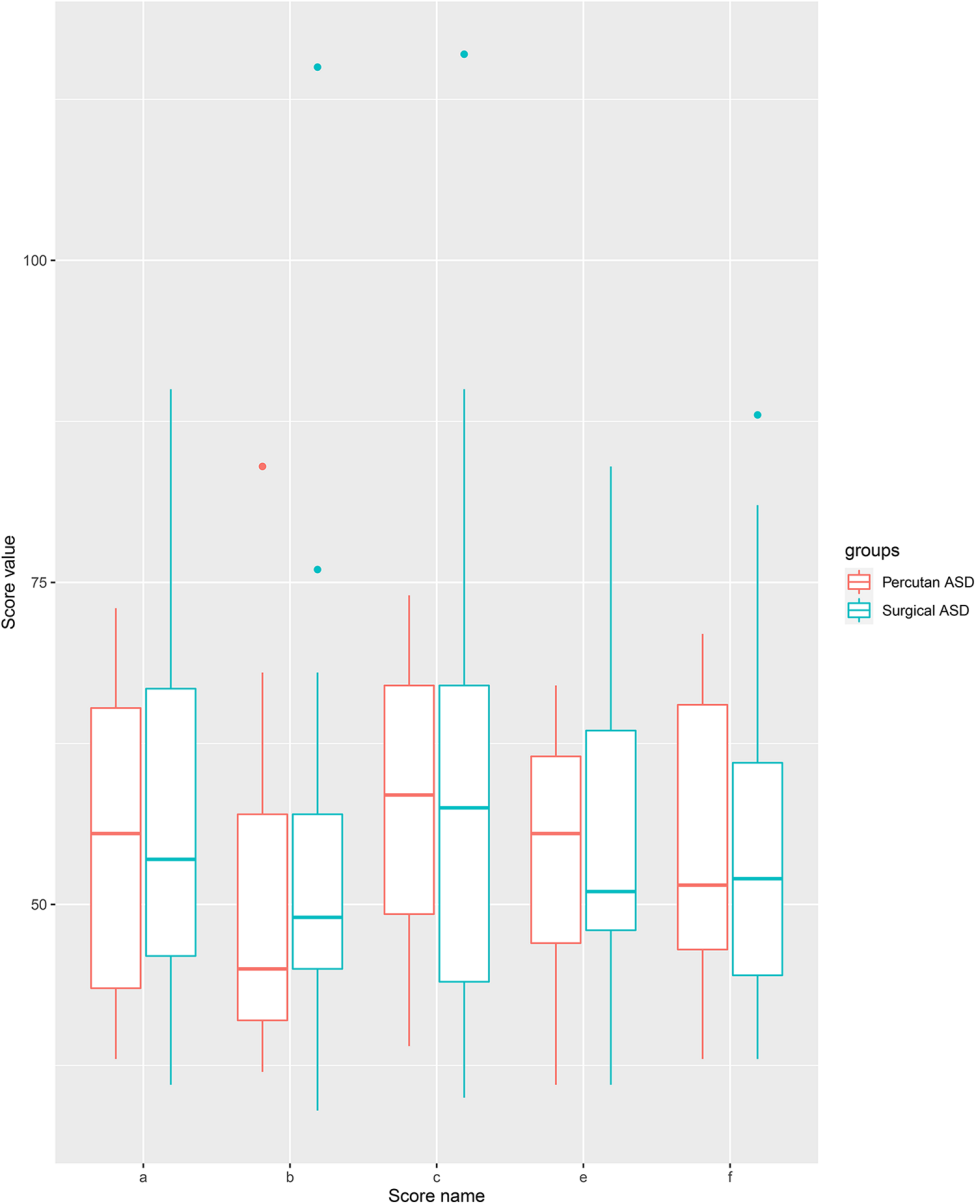
Boxplot of SRS-2 scores between surgical and percutanous closed ASD. a: Total scoes, b: social awareness, c: social cognition, d: social communication, e: social motivation, f: restricted interest and repetitive behavior.

We found no difference in any score between the group of patients that had a surgically closed ASD and a percutaneously closed ASD (Total score: *p* = 1, Social Awareness: *p* = 0.8, Social Cognition: *p* = 1, Social Communication: *p* = 0.74, Social Motivation: *p* = 0.8, Limited Interest and Repetitive behavior: *p* = 1) (Boxplot, Figure 3).

### Proxy-report

We also compared the proxy report between the two groups (Table 4). The proxy report in the CHD group was higher regarding the Total Score, the sub-scores of Social Cognition and Limited Interest and Repetitive Behavior. We found no difference between the CHD-proxy report and the control-proxy report with regard to the sub-scores of, Social Awareness and Social Motivation.

**Table 4:** Proxy-reported SRS-2 scores.

	CHD (*N* = 74)	Control (*N* = 33)	CHD vs. control
Total *T*-score	48.5 (41.0–56.0)	43.0 (39.0–47.0)	0.05
Social awareness	49.0 (43.0–58.0)	43.0 (37.0–52.0)	0.2
Social cognition	49.0 (43.0–59.0)	43.0 (40.0–49.0)	0.05
Social communication	47.5 (43.0–57.0)	44.0 (40.0–48.0)	0.2
Social motivation	50.0 (43.0–60.5)	45.0 (39.0–55.0)	0.2
Limited interest and repetitive behavior	50.0 (44.0–59.5)	44.0 (40.0–48.0)	0.05

Benjamini-Hochberg correction, false discovery rate 0.05. Non-parametric median test.

### Self-report vs. proxy-report

We found no difference in Total Score and sub-scores between self-report and proxy-report in the control group (Table 5). In the CHD group, the patients scored themselves worse in the Total Score and the Social Cognition sub-score compared to how their proxy scored them (Table 6).

**Table 5:** Control group reported SRS-2 scores.

Controls	Self (*N* = 38)	Proxy (*N* = 33)	Self vs. proxy
Total *T*-score	45.5 (40.5–50.8)	43.0 (39.0–47.0)	0.1
Social awareness	45.0 (41.0–56.0)	43.0 (37.0–52.0)	0.7
Social cognition	47.0 (42.0–53.0)	43.0 (40.0–49.0)	0.8
Social communication	44.0 (39.0–51.5)	44.0 (40.0–48.0)	1.0
Social motivation	45.0 (40.0–50.0)	45.0 (39.0–55.0)	1.0
Limited interest and repetitive behavior	44.0 (38.0–79.0)	44.0 (40.0–76.0)	0.5

Non-parametric Wilcoxon test.

**Table 6:** CHD group reported SRS-2 scores.

CHD	Self (*N* = 80)	Proxy (*N* = 74)	Self vs. proxy
Total *T*-score	52.5 (45.0–62.3)	48.5 (41.0–56.0)	0.02
Social awareness	49.0 (41.0–58.0)	49.0 (43.0–58.0)	0.5
Social cognition	55.0 (44.0–67.0)	49.0 (43.0–59.0)	0.01
Social communication	52.5 (44.0–63.0)	47.5 (43.0–57.0)	0.5
Social motivation	50.0 (45.0–60.5)	50.0 (43.0–60.5)	0.8
Limited interest and repetitive behavior	53.0 (38.0–98.0)	50.0 (40.0–96.0)	0.5

Non-parametric Wilcoxon test/non-parametric median test.

## Discussion

We found that young adults with a simple CHD (atrial or ventricular septal defect), had more social interaction difficulties than a matched control group. Our finding is in line with previous findings in children with more complex CHD (11, 13, 14, 17, 19). Social interactions and relationships are important throughout life as we live in a close social society (20, 21). Social difficulties have a negative correlation with socioeconomic status and personal income (22). We have previously found a higher risk of being on social security benefits in adults with simple CHD (10) and we speculate if their social interaction difficulties may play a role. Social difficulties (as in high functioning Autism Spectrum Disorder) affect all aspects of life, both in school and in the workplace (23, 24) leading to a potentially greater risk of being unemployed.

We found that one-third of patients with a simple CHD had a Total Score above 60, representing clinically significant difficulties with social interactions and a fourfold relative risk compared to the control group. Werninger et al. found a similar result in a group of children with more complex types of CHD (14). In our study, one-third of the patients with a simple CHD had an abnormal Total Score, and 3.6% had an abnormal Total Score above 85. This is in alignment with what we know about the participants where 42.5% had a psychiatric diagnosis and 3.8% had a diagnosed Autism Spectrum Disorder. A Total Score above 60 is related to social interaction difficulties and other clinical psychiatric diagnoses (e.g., ADHD and anxiety) and a Total Score above 85 is related to social interaction difficulties in Autism Spectrum Disorder (12). This alignment is in contrast to a pattern that we have previously seen in patients with CHD and symptoms of hyperactivity and inattention (8) and a pattern that is also seen in other chronic somatic disorders where more participants report symptoms of psychiatric diseases than how many are actually diagnosed (25, 26). Social interaction difficulties are seen in other psychiatric disorders such as ADHD (27). We found that a large portion of the variation in Total Score is explained by previously diagnosed psychiatric diseases. We also found a trend towards an increase in Total Score with an increase in the number of diagnosed psychiatric diseases. On one hand, psychiatric morbidities increase the risk of social interaction difficulties (18, 28). On the other hand, difficulties in social behavior increase the risk of social rejection (29) and social rejection in turn increases the risk of psychological difficulties (30). Our study design does not make it possible to determine the direction of the causality. In our study we find a good alignment with the SRS-2 Total Score cut-off and the number of participants that are diagnosed with a psychiatric disorder.

The participants with simple CHD scored similarly to the control group in the Social Awareness and in the Social Communication sub-scores. A previous study in children with more complex CHD found that these children had preserved social awareness but had trouble in the other areas of social interaction including social communication (14). The fact that we found a similar social interaction profile (with preserved social awareness) that was previously found in children with more complex CHD is noteworthy. It seems like patients with CHD both simple and complex defects can pick up social signals (Social Awareness) but have difficulties in other aspects of social interaction. This specific social interaction profile with preserved social awareness but difficulties in other areas of social interaction could increase the risk of depression (31) (they realize they are different but still have problems in social interactions). Older patients with a ventricular septal defect experience more often depression, lower health-related quality of life, and higher stress score (32). There could be different explanations as to why we do not see the same problems with social communication that was found in children with more complex CHD. One explanation could be that the CHD diagnoses in the cohort of children were worse thereby increasing the risk for neurodevelopmental difficulties in general (33) and also affecting social interaction. Another explanation could be that with the preserved social awareness in childhood, patients with CHD increase their ability to or learn to communicate better in social settings with age.

The proxies to the CHD participants reported the CHD participants to have more difficulties with social interaction in general (higher Total score) and specifically more difficulties with Social Cognition (higher Social Cognition sub-score) and with Limited Interest and Repetitive Behavior. These results tell us that the social interaction difficulties experienced by the CHD group are also to some degree evident to the surrounding world. On the other hand, we found that the participants with CHD reported themself to have more social interaction difficulties (higher Total score) and more specific difficulties in social cognition (higher Social Cognition sub-score) compared to how their proxy reported them. The fact that some psychological difficulties are hidden from even a close relative is something we have found in previous studies (8, 34). The Social Cognition sub-score reflects one's ability to accurately comprehend and interpret social cues correctly, making it challenging for the surrounding world to directly perceive the difficulties. These challenges manifest indirectly when the individual, experiencing the difficulties reacts in an “awkward” manner within a specific social situation. We do not find the same difference between the self- and proxy reports in the control group. We speculate that the CHD participants may compensate for their social interaction difficulties through their retained social awareness and social communication skills. As a result, they may appear more socially adept than they truly are. Anyway, the above-mentioned results could indicate that in young adults the self-report of “internal” problems, like social cognition, should have a greater saying than the proxy report.

The etiological pathway to social interaction difficulties is not clear. Social interaction involves many different functions in the brain (31). In Autism Spectrum Disorder, where difficulties in social skills play a large and important part, genetics, immunological dysregulation, metabolic disturbances, and early brain injury (both developmental and structural lesions) are suggested etiological pathways (31). Some of the same mechanisms are found to be affected in patients with CHD (35–39).

We found no difference between percutaneously closed ASD patients and surgically closed ASD patients. Bypass time and hospital length are short when dealing with simple CHD and may not predict social interaction difficulties. The results should be interpreted with caution as we are limited by the number of patients in each group (percutaneous ASD = 10, surgical ASD = 34). Our findings are backed by a previous study showing no association between surgical factors and social interaction in patients with complex CHD (14).

Social interaction is affected through many different pathways and one solution is probably not sufficient. Behavior therapy has the potential to enhance social skills in Autism Spectrum Disorder (40), and could maybe be used to enhance social skills in general.

### Strengths and limitations

To our knowledge, this is the first investigation of a social interaction profile in a group of patients born with a simple CHD (either atrial septal or ventricular septal defect). The profile that we found in this patient group is in line with the profile found in children born with more complex CHD. A notable strength in this study is that we included a control group matched on age, sex, and educational level, instead of only comparing the CHD group to normative data from the SRS2-manual.

There are limitations to our study. We excluded participants with a known syndrome. We are, of course, limited by the age of the participants and the tests available at the time of diagnosis. This means that the exclusion of genetic anomalies is relatively superficial by today's standards. Both the CHD group and the control group have an overrepresentation of women, which could affect the social interaction difficulties found and especially the profile found in this study. As the difference between sex in the Total score for adults are small [0.08 listed in the SRS-2 manual (12)], and both groups are comparable in sex distribution, we believe that the difference we have found between the two study groups are not due to most of the participants being women, but the specific social interaction profile could be different if the sex distribution was different. We investigated the social interaction profile from a questionnaire and not from a clinical examination. To compensate for this, we included a proxy report, but as illustrated in the results and discussion of this paper the more internal symptoms and the degree of social interaction difficulties are not easy to notice for even a close relative. Many factors are known to influence parental ratings, e.g., stress. Unfortunately, it was not possible to obtain that information. As participants are young adults, we have chosen to use the participants' own educational level instead of the parental educational level. As social interaction and cognition are developed during childhood and adolescence, parental educational level could influence the result and therefore would have been an important factor to have included in the study. Different factors can influence the scores in the SRS-2 and social interaction in general, for example (but not limited to) behavior problems, expressive language problems, and working memory (14, 17, 18). Because of collinearity between these factors and the factors used in the regression analysis, we did not include these otherwise relevant factors. We found that the participants with an already-diagnosed psychiatric disorder had more social interaction difficulties than the participants without a psychiatric diagnosis. It would be very interesting to know if these social interaction difficulties were present before more specific psychiatric symptoms or if they were developed after the psychiatric symptoms. This is not a question we can answer with our study design.

## Conclusion

We found that young adults with a simple CHD such as atrial and ventricular septal defects have a fourfold increased risk of social interaction difficulties than heart-healthy peers. They have a similar social interaction profile, with difficulties in social cognition and social motivation and preserved social awareness, as found in a group of children with more complex CHD, but unlike the children, the young adult with simple CHD also had preserved social communication. Problems with interpreting and understanding social cues correctly are especially difficult for the surrounding world to notice, making the self-reporting of internal problems highly valuable. Psychiatric morbidity explained most of the variation in social interaction problems. As social difficulties and psychiatric morbidities are intertwined, social interaction difficulties could be an indication of already underlying psychiatric morbidities or a risk factor for future psychiatric morbidity.

## Data Availability

The original contributions presented in the study are included in the article/Supplementary Material, further inquiries can be directed to the corresponding author.
